# Ochratoxin A and Kidney Oxidative Stress: The Role of Nutraceuticals in Veterinary Medicine—A Review

**DOI:** 10.3390/toxins14060398

**Published:** 2022-06-09

**Authors:** Consiglia Longobardi, Gianmarco Ferrara, Emanuela Andretta, Serena Montagnaro, Sara Damiano, Roberto Ciarcia

**Affiliations:** 1 Department of Mental, Physical Health and Preventive Medicine, University of Campania “Luigi Vanvitelli”, Largo Madonna delle Grazie n.1, 80138 Naples, Italy; consiglia.longobardi@unicampania.it; 2 Department of Veterinary Medicine and Animal Productions, University of Naples Federico II, Via Federico Delpino n.1, 80137 Naples, Italy; gianmarco.ferrara@unina.it (G.F.); emanuela.andretta@unina.it (E.A.); semontag@unina.it (S.M.); rciarcia@unina.it (R.C.)

**Keywords:** OTA, nutraceuticals, feed supplementation, oxidative stress, food safety, kidney

## Abstract

The problem of residues of toxic contaminants in food products has assumed considerable importance in terms of food safety. Naturally occurring contaminants, such as mycotoxins, are monitored routinely in the agricultural and food industries. Unfortunately, the consequences of the presence of mycotoxins in foodstuffs are evident in livestock farms, where both subacute and chronic effects on animal health are observed and could have non-negligible effects on human health. Ochratoxin A (OTA) is a common mycotoxin that contaminates food and feeds. Due to its thermal stability, the eradication of OTA from the food chain is very difficult. Consequently, humans and animals are frequently exposed to OTA in daily life. In this review article, we will devote time to highlighting the redox-based nephrotoxicity that occurs during OTA intoxication. In the past few decades, the literature has improved on the main molecules and enzymes involved in the redox signaling pathway as well as on some new antioxidant compounds as therapeutic strategies to counteract oxidative stress. The knowledge shown in this work will address the use of nutraceutical substances as dietary supplements, which would in turn improve the prophylactic and pharmacological treatment of redox-associated kidney diseases during OTA exposure, and will attempt to promote animal feed supplementation.

## 1. Introduction

Ochratoxin A (OTA), or *R*-*N*-[(chloro-3,4-dihydro-8-hydroxy-3-methyl-1-oxo-1H-2benzopyran-7-yl) carbonyl]-phenylalanine, is the most common mycotoxin among the ochratoxins, and it is mainly produced by some toxigenic filamentous fungi of species of the genera *Aspergillus* (*A.*) and *Penicillium (P.)*, such as *A. ochraceus, A. niger*, *A. carbonarius*, and *P. verrucosum*. In particular, in tropical areas, it is mainly produced by *A. ochraceous*, while in tempered regions it is mainly produced by *P. verrucosum* [1].

From a chemical point of view, OTA is a pentaketide with a very stable dihydrocoumarin linked to an L-β-phenylalanine residue. It occurs in a colorless crystalline form, and is soluble in polar organic solvents as well as being slightly soluble in water [2]. Being a weak organic acid, it acquires an intense green fluorescence when exposed to UV radiation in an acidic medium and a blue fluorescence when in an alkaline one [3].

OTA is a common contaminant of several feedstuffs and raw agricultural commodities, since it is not destroyed by standard food preparation procedures. Its hazardous nature comes from its great stability, as it is highly resistant to high temperatures and highly acidic environments. The thermostability of OTA is demonstrated by its partial degradation at normal cooking temperatures and after sterilization with saturated steam at 121 °C for 3 h [4,5]. Due to its extreme stability and its almost ubiquitous occurrence as a food contaminant, there is a high risk of intake of OTA in the diet [6]. OTA is commonly detected in coffee, cacao, grapes, raisins, wine, soy, spices, nuts, pulses, liqueurs, and beer; it is estimated that contaminated cereals (contaminated before and after harvest) and cereal products account for 60% of this exposure [7,8]. A significant amount of OTA may also be present in animal-derived food items, as it can accumulate in the meat and organs of these animals because of its significant occurrence in the feed of livestock [9]. 

Heussner et al. described the frequency of the occurrence of OTA in various feeds. In multiple surveys conducted in different European countries (barley, wheat, oats, and maize), high contamination of cereals with OTA was found (between 15% and 48%). As a result of this contamination (which affected up to 100% of the feed samples), the transfer of these substances into meat and derivatives is a logical conclusion. This aspect is relevant to pork (the meat and organs of which are contaminated at high percentages), which, together with cereals, is the main cause of human exposure to OTA as well as economic losses in the swine industry [10]. Since OTA is classified as a Group 2B carcinogen (IARC—International Agency for Research on Cancer, 1993), legislative authorities felt compelled to propose legislation to control OTA in food and feedstuff commodities because of the high risk associated with the potential transfer of OTA from feed to animal tissues and food products. The European Commission has established some guideline levels for the acceptability of feed and products for animal nutrition (Table 1) [11]. In the USA, the FDA (Food and Drug Administration) has not set OTA limits for these products, even though mycotoxin is included in the list of potentially hazardous ingredients in animal feed and feed ingredients.

The kidney is the main target of OTA exposure, which causes ochratoxicosis in poultry [12], and it can provoke mycotoxic porcine nephropathy in pigs [13]. The clinical signs of mycotoxic porcine nephropathy cannot be observed earlier than when pigs are slaughtered, because the kidneys appear pale and enlarged. A histological examination shows the degeneration of the proximal tubules followed by the atrophy of the tubular epithelium, glomerular hyalization, and interstitial fibrosis at the renal cortex level [14]. Both turkeys and chickens show reduced productivity during ochratoxicosis outbreaks, and symptoms include retarded growth, reduced feed conversion, and nephropathy, which even lead to death. Moreover, affected animals are apparently more susceptible to air sacculitis inflammation caused by *Escherichia coli* (CAST—Council for Agriculture Science and Technology, 2003) [15]. Therefore, the European Food Safety Agency (EFSA) concluded that OTA is a potent renal toxin in animals, such as poultry and pigs [16].

According to animal studies in murine models, OTA-induced renal toxicity and genotoxic effects are most likely mediated by the formation of free radicals, which can lead to kidney cancers (EFSA, 2006). The generation of reactive oxygen species (ROS) is one of the key cellular processes that cause OTA-induced kidney damage, even if the molecular mechanisms behind its effects are unknown as of yet [17].

Many veterinarians, nutritionists, food scientists, and animal health professionals recognize the importance of nutraceuticals in animal health and diseases [18]. Stephen De Felice was the first to coin the term nutraceutical, composed of two words: “nutrition” (nourishing element) and “pharmaceutical” (medicinal component). According to its inventor, a nutraceutical is “a food (or part of a food) that provides medical or health benefits, including the prevention and/or treatment of a disease” [19]. 

In this review, we have analyzed substances of a natural origin that are capable of reducing oxidative stress on the kidneys, and, consequently, how an intervention in this organ can prevent serious damage due to OTA intoxication.

### OTA’s Toxicokinetic Properties and Their Consequences in Animal Production

The toxicokinetic properties of OTA were species-specific, although it is rapidly absorbed in all animal species and the elimination half-life is very long. Because of its strong affinity for proteins, especially those with a molecular weight of 20 kDa, it tends to accumulate in humans and animals. In fact, OTA binding proteins have been found in the plasma of several animal species, as well as in humans. This property favors its accumulation in the vascular system, the liver, and muscles [20]. In addition, OTA has been proven to inhibit the adipogenic differentiation of human mesenchymal stem cells (hMSCs) isolated from adipose tissue [21]. 

Products of an animal origin therefore contribute to the human intake of OTA. It is present in processed meat products, such as muscles, offal, milk, and eggs, but the highest concentrations are detected in cured meats, blood sausages, pậtés, and sausages. This phenomenon promotes bioaccumulation in the organs of animals, leading to the contamination being carried over [22,23].

Monogastric species absorb OTA from the gastrointestinal tract without prior degradation. In particular, in broiler chickens, accumulation of OTA, principally in the kidney and liver, leads to a decrease in egg production and weight loss after the administration of OTA-contaminated feed [24].

Ruminants are less sensitive to OTA-induced toxicity because the rumen produces fewer toxic metabolites, such as OTα (ochratoxin α) [25]. However, following an alteration in the feed composition, e.g., an increase in the protein content, which may modify the hydrolytic capacity of rumen microorganisms, the ability to detoxify OTA is impaired. Although the OTA content in ruminant milk seems to be very low, the OTA occurrence in dairy products, such as cheese, may be higher [26,27]. An interesting study on cave cheese, a surface-mold-ripened cheese produced in Southern Italy, revealed the presence of ochratoxigenic species, *A. westerdijkiae* and *A. steynii,* and suggests a possible mycotoxin risk during cave ripening, confirmed by Anelli et al., who demonstrated the presence of OTA in a significant group of samples [28].

The toxicokinetic properties of OTA have also been studied in fish, such as Nile tilapia [29]. Recent studies on Atlantic salmon have confirmed that only small traces of OTA enter the edible tissue, allowing fish to be considered as a nonhazardous source of OTA exposure [30].

In this scenario, the search for natural sources administered to animals to prevent OTA contamination could open the door to new health and economic perspectives.

## 2. OTA Mechanisms of Kidney Toxification: Focus on Oxidative Stress

As with most mycotoxicosis, OTA often leads to chronic intoxication due to a prolonged intake over time. The toxic effect mainly affects the kidneys, but other organs, such as the liver and the immune system, are also affected [31,32,33,34]. Some studies carried out in laboratory animals have shown that renal cancer is most common with OTA exposure [35].

In vitro studies on liver and kidney microsomes, as well as in vivo experiments in rats, have shown that OTA enhances NADH (nicotinamide adenine dinucleotide)-dependent and ascorbate-dependent lipid peroxidation. They proposed that OTA stimulates both NADH-dependent and ascorbate-dependent lipid peroxidation in microsomes, using iron (Fe^3+^) as a cofactor. An OTA–Fe^3+^ complex would be formed, which would facilitate the reduction of Fe^3+^ in the presence of an NADH–cytochrome P450 reductase system. The resulting OTA–Fe^2+^ complex would then generate OH•, leading, in turn, to membrane lipid peroxidation [36]. A study conducted by Khan et al. showed that lipid peroxidation enhanced by OTA impairs cytoplasmatic membrane permeability to calcium (Ca^2+^), thus affecting Ca^2+^ homeostasis by increasing its influx; this ion is released from the intracellular stores and affects the sensitivity of Ca^2+^ channels [37]. It has been reported that the administration of a single high dose or multiple lower doses of OTA to rats resulted in an increase in the activity of the ATP-dependent Ca^2+^ pump in the ER (endoplasmic reticulum) of renal cortex cells. Thus, OTA could interfere with all of the cellular functions under the control of calcium concentration [38]. Many studies have suggested that intracellular Ca^2+^ accumulation could be considered a common step in the development of OTA cytotoxicity, since the alteration of Ca^2+^-regulated mechanisms is often an early event in the development of cellular damage. Both an increase in cellular Ca^2+^ concentration and the presence of OTA could participate in the uncoupling of the oxidative balance and, consequently, in the production of ROS [20,39,40]. Furthermore, Damiano et al. demonstrated, in a rat model treated for 2 weeks with OTA, that an increase in ROS production was also linked to a strong reduction in the glomerular filtration rate (GFR) and a significant increase in blood pressure [41]. 

During OTA exposure, some enzymatic pathways involved in the metabolism of an excessive amount of ROS are altered. The best-characterized adaptive pathway controlling the antioxidant response in mammalian systems is the nuclear factor E2-related factor 2 (Nrf2)/ARE signaling pathway [42]. Nrf2 is responsible for both basal and adaptive antioxidant levels in response to oxidative stress. In basal conditions, Nrf2 is bound to the endogenous inhibitor Kelch-like ECH-associated protein 1 (KEAP1). Once activated, it migrates to the nucleus and binds to ARE. The binding of Nrf2–ARE regulates the expression of more than 200 genes involved in cellular antioxidant and anti-inflammatory defense [42]. The transactivation of Nrf2, one of the major contributors to OTA-induced oxidative stress, is impaired in cultured porcine kidney tubule cells (LLC-PK1) treated with OTA, and the reduced ratio of GSH (glutathione)/GSSG (glutathione disulfide, GSH-oxidized form) increases the amount of ROS [42].

The modulation of some cellular and molecular markers involved in cellular proliferation and growth, cell death, cell survival, cellular function, and maintenance was observed in model organisms, such as pigs, by OTA toxicity [42]. Marin et al. showed that OTA can affect the expression of some genes involved in the oxidative stress response (Jun, SCARB1, MAF, and ACTG2), according to the Nrf2-mediated signaling pathway [43].

Antioxidant enzymes’, such as SOD (superoxide dismutase), CAT (catalase), and GPx (glutathione peroxidase), activities may be affected by OTA. The treatment of a pig kidney cell line (LLC-PK1) with increasing concentrations of OTA decreased SOD activity and increased the intracellular levels of ROS in a dose-dependent manner [42]. In a recent study, Garcia et al. found the downregulation of glucose-6-phosphate dehydrogenase (G6PD) and GPx mRNA levels in LLC-PK1, whereas CAT and SOD were upregulated in human kidney cells (HK-2) after being exposed to OTA [17]. An in vivo toxicity study on the relationship between OTA and oxidative stress in rats showed an increase in MDA levels in the kidney as well as the downregulation of GPx and SOD gene expression [44]. CAT and SOD activities in the kidney are unaffected in poultry, while pigs showed a decrease in GPx kidney activity [16,45].

In addition, high levels of ROS, leading to a decrease in the main cellular antioxidant enzymes responsible for free radical protection, promote the production of MDA (malondialdehyde), a lipid peroxidation biomarker, that damages several components of the cell [46]. MDA “sticks” the amino–protein groups and causes the formation of lipid–protein complexes that cannot dissolve (lipofuscin). The immune system cannot ignore these physiologically harmful formations, and so the inflammatory process begins [47]. Several pieces of data in the literature have shown the relationship between oxidative stress and inflammation, and there is evidence that oxidative stress plays a pathogenic role in chronic inflammatory diseases. Oxidative stress increases the level of proinflammatory cytokines and upregulates inflammatory molecules, such as vascular cell adhesion molecule-1 (VCAM-1), intercellular adhesion molecule-1 (ICAM-1), and nuclear factor-kappa B (NF-κB), in many pathogenic diseases, including during OTA intoxication [48].

OTA is also responsible for increasing the activity of iNOS as well as the expression and activity of dimethylarginine-dimethylaminohydrolase (DDAH), involved in nitric species homeostasis and promoting increased nitrogen monoxide (NO) synthesis, along with increased levels of RNS, i.e., nitrites and nitrates [49].

NADH oxidase (NOX) is involved in the regulatory processes of homeostasis and appears to be the most important contributor to ROS generation in the kidney [50,51]. 

The most abundant NOX isoform in the kidney is NOX4, called “renox” by Geiszt et al. [52]. NOX4 specifically increases O_2_− production in the mitochondria, is rapidly catalyzed to H_2_O_2_, and diffuses into cells to promote apoptosis [53]. Moreover, it is well-established that the downregulation of NOX4 reduces oxidative stress by decreasing its own O_2_− production and inhibiting mitochondrial dysfunction [53]. Transgenic mice that overexpressed NOX4 developed increased levels of oxidative stress, increased myocardial apoptosis, and, furthermore, cardiac dysfunction [54]. According to its mechanism of action, it is involved in the regulatory processes of homeostasis, and seems to be the main contributor to ROS formation in the kidney [55,56,57]. Under physiological conditions, NOX activity is low, but in pathological ones its concentration increases and induces O_2_− formation. The excessive renal activity of NOX4 is considered one of the crucial factors for the progress of some oxidative-stress-associated diseases, such as diabetic nephropathy, and its inhibition is widely discussed as a promising new target for therapeutic strategies [50].

**Figure 1:**
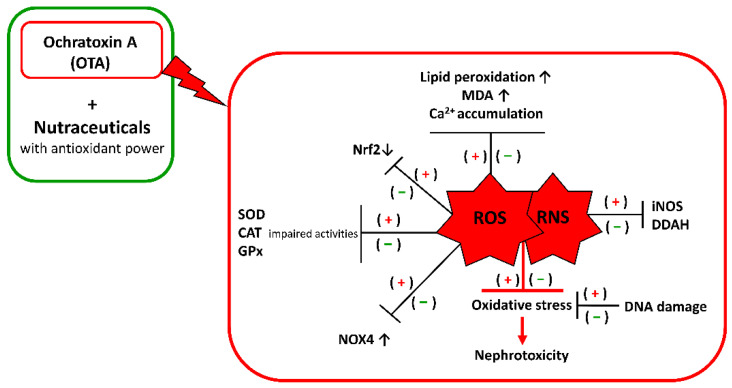
Schematic representation of how nutraceuticals can contribute to food safety against OTA intoxication. The (+) symbol indicates an increase in the oxidative effects due to OTA alone; the (−) indicates a decrease in the oxidative effects due to the addition of nutraceuticals with antioxidant power.

The evidence for oxidative stress in controlling OTA adverse effects argues for protection by compounds with the ability to scavenge free radicals, such as nutraceuticals with antioxidant power (Figure 1).

## 3. Nutraceuticals against OTA Kidney Oxidative Stress: An Additional Value in Veterinary Medicine

Nutraceuticals are nontoxic, renewable, and readily available products characterized by a certain ease of degradation and storage. These characteristics could provide a great advantage in the use of natural molecules to improve animal health and, consequently, that of humans [58]. 

Nutraceuticals contain many bioactive multitarget compounds, and, thanks to this property, have a wide application in many animal and human diseases. They can exert both biological and pharmacological effects, such as protection against free radicals and antioxidant effects [18]. 

As mentioned above, lipid peroxidation, which is considered to be one of the most important consequences of mycotoxicosis, is associated with the generation of free radicals, whose imbalance causes functional and structural changes in the kidney, the main organ responsible for the metabolism of OTA [59].

Numerous studies highlight that OTA-induced nephrotoxicity and carcinogenicity may be consequences of the formation of ROS, such as superoxide anion (O_2_−), hydroxyl radicals (OH–), and peroxide (ROO–), as they cause significant lesions to cellular structures and impair antioxidant cell defenses [60].

Given the important role of oxidative stress in a kidney subjected to OTA intoxication, several antioxidants have been used for their beneficial effect in regulating ROS/RNS production and neutralization, as well as maintaining balance.

In this context, nutraceuticals with antioxidant activity could represent important examples of natural substances that can be added to feeds assumed to be contaminated by OTA. Today, the scientific community, including veterinarians, have recognized the importance of nutraceuticals in animal health, and many studies have tested their effectiveness in preventing and protecting against OTA nephrotoxicity and oxidative stress, as they are some of the major mechanisms of action of this toxin [61]. 

This chapter focused on antioxidant systems that could be important in preventing and mitigating the adverse effects of ROS and RNS imbalance during OTA exposure. The following table (Table 2) will propose some nutraceuticals that could be used as feed supplements. 

### 3.1. Curcumin

Among the natural remedies studied, curcumin (CURC) has attracted the attention of the scientific community for its medicinal potential, confirmed by numerous pharmacological effects reported in scientific research [74].

CURC is a phenolic compound isolated from *Curcuma longa* of the Zingiberaceae family. Due to its chemical reactivity, resulting from an extended conjugated double-bond system susceptible to nucleophilic attacks, CURC interacts with numerous molecular targets as a potential therapeutic agent against several chronic diseases. Additionally, it is recognized for its antioxidant properties and used as a medical plant worldwide, especially in Asian countries [75].

In vivo studies have proven CURC’s therapeutic effects on renal function. In a rat model reproducing renal oxidative damage caused by OTA administration, CURC was administered. In this way, CURC attenuated the alterations in the activities and proved its ability to mitigate OTA-induced oxidative damage in the kidneys of rats [76]. CURC exerted cytoprotective and antioxidant activities against OTA-induced toxicity in rats by regulating inflammation and oxidative stress enzymes as well as reducing NO levels and oxidative DNA damage in the kidney, in addition to liver tissues [62].

### 3.2. Red Orange and Lemon Extract

Red orange and lemon extract (RLE) is obtained by appropriately mixing anthocyanins and other polyphenols obtained from red orange processing wastes together with erythrocin and other flavanones recovered from lemon peels [62].

Because of its antioxidant characteristics, RLE was tested on Sprague Dawley rats poisoned with OTA, whose nephrotoxicity has been shown to be related to oxidative stress. The coadministration of RLE reduced MDA concentration in the kidneys, and this could be due to the pronounced antioxidant properties of the compounds contained in the extract. In addition, the kidney tissues of rats treated with RLE exhibited less severe fibrosis than those of OTA-intoxicated rats [77]. This condition also suggests the protective role of RLE in the development of fibrosis and confirms its potential to counteract oxidative stress in the kidneys. 

Thus, the addition of RLE to feed could reduce OTA toxicity in animals and, consequently, toxicity in humans after consumption. In this regard, Damiano et al. further investigated the mechanism of action and proved RLE’s ability to prevent renal damage during the progression of nephropathy by inhibiting the increase in NOX4 [78]. In a recent article, RLE was suggested as a dietary supplement to protect lambs from oxidative stress [79], supporting the idea of considering RLE as a dietary feed supplement to counteract the adverse effects of OTA poisoning. 

### 3.3. δ-Tocotrienol

Tocotrienols are fat-soluble molecules, commonly referred to as vitamin E, that could have beneficial effects against OTA toxicity by acting as free radical scavengers. Among them, δ-tocotrienol has been tested in male Sprague Dawley rats treated with OTA. In this study, the increase in renal oxidative stress induced by OTA was reversed by concomitant treatment with δ-tocotrienol. Indeed, MDA levels were modulated by δ-tocotrienol along with SOD, CAT, and GSH enzyme activities, confirming the role of ROS in the development of kidney diseases and demonstrating its potential use as a dietary supplement to protect organisms from OTA-induced damage [41]. This research has provided a new basis for the protection of animals, their edible products, and, by extension, humans.

### 3.4. Quercetin

Quercetin belongs to flavonoid compounds. Because it can scavenge highly reactive species, such as peroxynitrite and hydroxyl radicals, it may have beneficial health effects on pathologies associated with oxidative stress. Numerous studies have been performed to gather scientific evidence for these health-promoting claims and demonstrated that quercetin, with its Fe^3+^-chelating properties, is one of the most prominent dietary antioxidants [80].

In a study by Lu et al., quercetin was shown to be able to inhibit M1 macrophage polarization via NF-κB and IRF5 (interferon regulatory factor 5) signaling, thereby ameliorating renal injuries. Interestingly, quercetin also inhibits M2 macrophage polarization and reduces the excessive accumulation of the extracellular matrix via the TGF-β (transforming grow factor β)/Smad signaling pathway. As a result, interstitial fibrosis in the kidneys is reduced by quercetin treatment. Thus, quercetin has therapeutic potential for patients with kidney injuries [81].

However, treatment with quercetin in combination with chitosan nanoparticles (COS-NPs) offers a reasonable way to improve the efficacy and oral bioavailability of quercetin as well as the therapeutic effect of chitosan itself [82]. In fact, COS-NPs plus quercetin showed higher protection against OTA-induced cytotoxicity than quercetin alone [63]. The successful completion of this treatment means that COS-NPs plus quercetin may be considered as a possible candidate for feed protection to counteract the health hazards of OTA.

### 3.5. Apocynin

Apocynin is a dietary flavonoid and a potentially attractive oral prodrug because of its low general toxicity [83]. Its specific antioxidant activity is triggered after metabolic activation by myeloperoxidase with the release of phagocytic cells. Few data are available on the safety of apocynin, but those that are available show its low toxicity and high stability [84].

Data from renal studies show that apocynin protects the kidney function from the toxic effects induced by cyclosporin A through the inhibition of NOX activity, with the recovery of O_2_− and NO production to the balance values, because of NOX blockage [85].

Additionally, the administration of apocynin to a rat model of diabetes mellitus prevents the translocation of p47phox to the plasma membrane of the kidney, and, consequently, inhibits NOX4 by preventing the assembly of its p47phox and p67phox subunits to form a NOX complex [86].

Sheu et al. proved the efficacy of apocynin as a NOX inhibitor in glomerular mesangial cells of mice and rats treated with OTA. In fact, apocynin significantly inhibited OTA-induced mesangial cytotoxicity and apoptosis, demonstrating its role in preventing OTA intoxication [64].

### 3.6. Catechins

Catechins belong to a group of flavanols present in green tea leaves, chocolate, and some plants, and boast anticancer as well as antioxidant properties [87]. Their capacity to restrict free radical formation, scavenge free radicals, and chelate transition metal ions, notably Fe and Cu, which act as catalysts in free radical reactions, exemplifies their antioxidant capacities [88].

These compounds include epigallocatechin gallate (EGCG) and epicatechin gallate (ECG), two molecules tested in vitro by Costa et al. on an LLC-PK1 cell line treated with increasing concentrations of OTA. The results obtained by this research group showed that the pretreatment of cells with EGCG and ECG has a preventive function by reducing the intracellular ROS concentration, DNA fragmentation, and apoptosis induced by OTA. The hypothetical mechanism attributed to them is ROS chain breaking, particularly at pH = 2, which encourages prevention through feed supplementation as a suitable strategy to reduce OTA toxicity as well as those caused by other mycotoxins [65].

### 3.7. Salvia Farinacea and Azadirachta Indica Water Extracts

Since OTA intoxication derives from the proliferation of fungi of *A. ochraceous* and *niger* species in meats, such as sausages, beef burgers, etc., it may be advisable to target this mold with water extracts of *Azadirachta indica* (Meliaceae) and *Salvia farinacea* (Lamiaceae). The first is also known as the neem tree and contains numerous phytochemicals, such as catechins, flavonoids, tannins, quercetin, and gallic acid, to name a few, while the second one is mainly rich in flavonoids and phenolic acid [89,90].

Hamad et al. used a combination of these two extracts in a ratio of 1:1 *v*/*v*, which showed the highest antifungal activity in vitro against neem and mealy cup sage alone on the growth of both fungi mentioned above, while reducing OTA production. The same extract was also tested in vivo on albino rats, demonstrating the safety of this combination in protecting the kidneys from OTA toxicity [66]. This kind of extract may have a dual application: inhibiting fungal growth and extending the shelf life of meat products.

### 3.8. Cyanidin-3-O-β-glucopyranoside

Anthocyanins, secondary metabolites found in fruit and vegetables, and responsible for their pigmentation, have recently stimulated a considerable interest as powerful antioxidants, capable of preventing oxidative damage caused by ROS [91,92]. The biological role of anthocyanins in the plant world is to attract insects and contribute to pollination and seed dispersal [93]. 

Among anthocyanins, cyanidin-3-*O*-glucopyranoside (C-3 g) is the one mainly found in blood oranges, but also in blueberries, strawberries, currants, and pomegranates, and it has a particular antioxidant effect due to it having numerous phenolic groups. Part of its mechanism of action is attributed to its ability to chelate divalent metal ions, which are required for ROS generation by the Fenton reaction. Amorini et al. have shown that C-3 g acts as a true antioxidant due to its peculiar redox potential. All of the C-3 g concentrations tested were effective in inhibiting MDA formation and the lipid peroxidation index in vitro [94].

Additionally, the group of Guerra et al. demonstrated the antioxidant effect of C-3 g on the OTA cytotoxicity induced on the Hep G2 and CaCo-2 cell lines. Pretreatment with C-3 g for 24 h significantly prevented OTA cell damage in both cell lines [67], proving to be a promising antioxidant for future studies.

### 3.9. Luteolin and Hydroxytyrosol

Luteolin (LUT), a flavonoid found in *Matricaria chamomilla*, exhibits high bioavailability as well as strong recovery and antioxidant properties, in contrast to the other flavonoids [95]. In NRK-52E rat kidney cells, LUT reduces ROS formation, ameliorates the reduction in the mitochondrial membrane, and restores the activity of antioxidant enzymes to control levels, thereby alleviating OTA-induced oxidative stress. The mechanism behind the effects of LUT is the activation of the Nrf2 pathway, which increases the antioxidant defense capacities of cells treated with OTA [68]. 

Hydroxytyrosol (HT) is a plant chemical compound found in olive oil that, together with oleocanthal, is responsible for the slightly bitter and spicy taste of extra virgin olive oil. In a recent study by Crupi et al., the effect of HT on OTA-induced renal injury was investigated both in vitro and in vivo. Rats administered OTA had higher levels of MDA and lower levels of nonenzymatic antioxidants, such as SOD, GSH, and CAT, than control animals [69].

These results strengthen the hypothesis that LUT and phenols from olive oil, such as HT, could help to reduce the burden on the kidneys from the development of OTA oxidative stress and be used as a dietary supplement in feed.

### 3.10. Marine Algae

Nowadays, the marine ecosystem is considered as an ideal reservoir of new molecules and the development of marine nutraceuticals [96]. Therefore, it has earned the title of “Natural Medicine Chest of the New Millennium”, and is also becoming an important purchasing power worldwide [97,98]. In fact, the use of marine organisms as natural sources of new substances that can contribute to human nutrition and overall health is increasing [99].

The importance of marine algae as a source of natural antioxidants could be a novelty in their therapeutic use: in this perspective, these compounds could be used against various diseases and ageing processes by protecting cells from oxidative damage [99], paving the way for a new therapeutic strategy against renal oxidative stress. 

Data presented by Nabil-Adam and Shreadah suggested that the marine extract of *Galaxaura oblongata* could have nutraceutical and pharmaceutical applications because of its promising therapeutic agents against LPS-induced acute kidney injuries through antioxidant and anti-inflammatory mechanisms of action, although further studies are required [70].

Marine carotenoids have also shown potential benefits, particularly astaxanthin and fucoxanthin [100]. Both carotenoids exhibited strong antioxidant activity by quenching singlet oxygen and scavenging free radicals. Furthermore, since a rise in Nrf2 prevents and treats a variety of chronic diseases, including those affecting the kidneys, carotenoids provide cellular protection against oxidative-stress-induced nephrotoxicity through the induction of Nrf2–ARE-mediated antioxidant enzymes [101]. Other carotenoids of this kind need to be evaluated for their potential as drugs and/or functional foods against OTA-induced oxidative stress in the kidney.

Another alga, called *Alsidium corallinum*, a red Mediterranean one, has shown antioxidant activity due to the presence of flavonoids and polyphenol. The corrective effect of this alga on potassium-bromate-induced kidney injury in vivo showed that this type of supplementation significantly prevented potassium-bromate-induced nephrotoxicity, as indicated by plasma biomarkers and OS-related parameters in renal tissue [71]. 

In addition, the study proposed by Yang et al. demonstrated the antioxidant activity and inflammatory response of *Ulva lactuca* polysaccharide extract (UPE) against oxidative stress induced in a D-galactose (D-gal)-induced ageing model in vivo [73]. In a complex, the results showed that UPE could significantly improve the activities of SOD and GSH-GPx, as well as the total antioxidant capacity in mice, which significantly ameliorated D-galactose-induced kidney injury [73].

Astaxanthin (ASX), also known as lobster shell, is a molecule from the carotenoid family extracted from *Haematococcus pluvialis*, a green alga known for its high antioxidant potential, the most powerful compared to all of the other carotenoids [72,102].

The group of Li et al. conducted a study on ASX’s ability to contrast OTA oxidative stress in the kidney. In their study, they focused on the Nrf2 pathway and demonstrated the in vivo efficacy of using ASX to treat OTA poisoning. Renal physiological parameters confirmed that the pretreatment of poisoned rats with ASX reduced the activity of SOD, CAT, and GPX compared with rats that did not receive ASX. Additionally, the mRNA and protein expression of Nrf2 were increased according to the literature, but in the presence of ASX, Nrf2 shifted from the cytoplasm to the nucleus and enhanced the expression of proteins involved in the redox balance, thus acting as a protector of kidney cells [103]. 

All of these findings about marine algae provide a basis for the development of antioxidants and the treatment of kidney diseases. The idea is to test whether seaweed products can also be used in OTA intoxication, since they are natural and considered as a new “functional food”. In fact, they could be used as a feed additive in animal production to reduce the risk of OTA intoxication or, in any case, to limit its adverse effects. 

## 4. Conclusions

The occurrence of OTA as a contaminant in agriculture is widespread throughout the world and causes adverse effects in higher organisms in both acute and chronic exposure [104].

OTA is known for its nephrotoxicity, hepatotoxicity, and immunotoxicity, and is harmful to the swine and poultry industries as well as to humans; in fact, it is known to contribute to the pathogenesis of Balkan endemic nephropathy (BEN) [105].

The mechanism of action of OTA is still unclear, and the development of strategies to alleviate the toxicity caused by OTA is very complex. In this review, we evidenced oxidative stress as a probable mechanism for OTA-induced toxicity. Reducing ROS production, activating the Nrf2 pathway, maintaining DNA stability, or using nanoparticles as adsorbents for mycotoxins or innovative marine algae are different ways to prevent OTA toxicity. 

Both humans and animals are inevitably exposed to OTA-contaminated foods. The revealed molecular mechanism of OTA nephrotoxicity is an important topic that has generated further opinions and strategies, such as prophylactic foods or feed additives, which could counteract OTA toxicity to protect human and animal health [106]. 

The development of effective strategies that alleviate OTA-induced nephrotoxicity is fueled by the need to reduce the toxic effects of this mycotoxin. Since its toxic effects are focused on oxidative stress, and interest in the nutraceutical sector continues to grow, the use of nutraceuticals with antioxidant activity could improve food and feed safety.

The oxidative stress machinery changes the perception that we have of molecular oxygen, and is now considered an undeniable pharmacological target in medicine. In recent years, new natural compounds, administered alone or in association with classical therapies, have been proposed as therapeutic agents against OS. 

In this review, we have described the effect of dietary supplements and some nutritional strategies on reducing the exposure to free radicals and increasing antioxidant intake or antioxidant supplementation intake to reduce OTA toxicity. 

Interest in the nutraceutical sector continues to grow, in part due to ongoing research to identify and characterize the use of additional compounds, as well as high consumer demand in the continuous search for “Functional foods” that guarantee immunity to various kinds of “endemic” diseases, including the toxicity induced by mycotoxins [107].

## Figures and Tables

**Table 1:** OTA main mold source and guidance values in mg/Kg (ppm) regarding products intended for animal feed with a moisture content of 12% set by the Commission Recommendation 2016/1319 of 29 July 2016 amending Recommendation 2006/576/EC in regard to deoxynivalenol, zearalenone, and ochratoxin A in pet food.

	*OTA Mold Source*			
	*Aspergillus ochraceus*	*Aspergillus niger*	*Aspergillus carbonarius*	*Penicillium verrucosum*
**Products for Animal Feed Contaminated by OTA**	**ppm**			
Cereals and cereals products	0.25			
Complementary and complete feedstuffs for pigs	0.05			
Complementary and complete feedstuffs for poultry	0.1			
Complementary and complete feedstuffs for cats and dogs	0.01			

**Table 2:** Possible nutraceuticals as feed supplements to counteract OTA poisoning.

Proposed Feed Supplement	In Vivo/In Vitro Model	Main Antioxidant Effect	References
**Curcumin (CURC)**	Sprague Dawley rats	Reduction in lipid peroxidation, DNA damage and nitrosative stress, as well as enhancement of antioxidant enzyme activity	Longobardi et al., 2021 [62]
**Red orange and lemon extract (RLE)**	Zucker diabetic fatty rats	NOX inhibition	Damiano et al., 2020 [50]
**δ-tocotrienol**	Sprague Dawley rats	Restoration of antioxidant enzyme	Damiano et al., 2018 [41]
**Quercetin**	Sprague Dawley rats	Activation of the Nrf2–ARE pathway	Abdel-Wahhab et al., 2017 [63]
**Apocynin**	MES-13 cells (MMCs) and primary rat mesangial cells (RMCs)	NOX inhibition	Sheu et al., 2017 [64]
**Catechins**	Pig kidney cell line (LLC-PK1)	Generation of an active concentration near and inside the membrane surface to scavenge ROS	Costa et al., 2007 [65]
***Salvia farinacea* and *Azadirachta indica* water extract**	Albino rats	Decrease in the bioavailability of OTA	Hamad et al., 2021 [66]
**Cyanidin-3-*O*-β-glucopyranoside**	Human epatoma cell line (Hep G2) and a human colonic adenocarcinoma cell line (CaCo-2)	Absorption and neutralization of free radicals, quenching singlet and triplet oxygen, and decomposing peroxides	Guerra et al., 2005 [67]
**Luteolin (LUT)**	NRK-52E rat kidney cells	Activation of the Nrf2–ARE pathway	Liu et al., 2020 [68]
**Hydroxytyrosol (HT)**	Madin–Darby canine kidney cell line (MDCK), a pig kidney cell line (LLC-PK1), and a rabbit kidney cell line (RK 13); Sprague Dawley rats	Preservation of lipid peroxidation	Crupi et al., 2020 [69]
***Galaxaura oblongata***	BALB/C mice	Reduction in LPS-induced acute kidney injuries	Nabil-Adam and Shreadah, 2021 [70]
***Alsidium corallinum***	Mice of a Swiss strain	Prevention of potassium-bromate-induced nephrotoxicity	Ben Saad et al., 2015 [71]
**Astaxanthin (ASX)**	C57BL/6 mice	Activation of Nrf2–ARE-mediated antioxidant enzymes	Ni et al., 2018 [72]
***Ulva lactuca* polysaccharide extract (UPE)**	Kunming mice	Glomerular filtration rate recovery	Yang et al., 2020 [73]

## Data Availability

Not applicable.
